# Nicotinic Acid Accelerates HDL Cholesteryl Ester Turnover in Obese Insulin-Resistant Dogs

**DOI:** 10.1371/journal.pone.0136934

**Published:** 2015-09-14

**Authors:** Jérôme Le Bloc'h, Véronique Leray, Hassan Nazih, Olivier Gauthier, Samuel Serisier, Thierry Magot, Michel Krempf, Patrick Nguyen, Khadija Ouguerram

**Affiliations:** 1 INRA, UMR 1280, Physiologie des Adaptations Nutritionnelles, F-44093 Nantes, France; 2 LUNAM University, Oniris, National College of Veterinary Medicine, Food Science and Engineering, Nutrition and Endocrinology Unit, Nantes, F-44307, France; 3 CRNH, West Human Nutrition Research Center of Nantes, CHU, Nantes, F-44093, France; 4 MMS—EA 2160—Mer Molécules Santé, IUML—Institut Universitaire Mer et Littoral—FR3473 CNRS, Nantes, France; 5 LUNAM University, Oniris, National College of Veterinary Medicine, Food Science and Engineering, Small Animal Surgery Department, Nantes-Atlantic, Nantes, F-44307, France; University of Basque Country, SPAIN

## Abstract

**Aim:**

Nicotinic acid (NA) treatment decreases plasma triglycerides and increases HDL cholesterol, but the mechanisms involved in these change are not fully understood. A reduction in cholesteryl ester transfer protein (CETP) activity has been advanced to explain most lipid-modulating effects of NA. However, due to the central role of CETP in reverse cholesterol transport in humans, other effects of NA may have been hidden. As dogs have no CETP activity, we conducted this study to examine the specific effects of extended-release niacin (NA) on lipids and high-density lipoprotein (HDL) cholesteryl ester (CE) turnover in obese Insulin-Resistant dogs with increase plasma triglycerides.

**Methods:**

HDL kinetics were assessed in fasting dogs before and four weeks after NA treatment through endogenous labeling of cholesterol and apolipoprotein AI by simultaneous infusion of [1,2 ^13^C_2_] acetate and [5,5,5 ^2^H_3_] leucine for 8 h. Kinetic data were analyzed by compartmental modeling. *In vitro* cell cholesterol efflux of serum from NA-treated dogs was also measured.

**Results:**

NA reduced plasma total cholesterol, low-density lipoprotein cholesterol, HDL cholesterol, triglycerides (TG), and very-low-density lipoprotein TG concentrations (*p* < 0.05). The kinetic study also showed a higher cholesterol esterification rate (*p* < 0.05). HDL-CE turnover was accelerated (*p* < 0.05) *via* HDL removal through endocytosis and selective CE uptake (*p* < 0.05). We measured an elevated *in vitro* cell cholesterol efflux (*p* < 0.05) with NA treatment in accordance with a higher cholesterol esterification.

**Conclusion:**

NA decreased HDL cholesterol but promoted cholesterol efflux and esterification, leading to improved reverse cholesterol transport. These results highlight the CETP-independent effects of NA in changes of plasma lipid profile.

## Introduction

The lipid-modulating effects of nicotinic acid (NA) were reported almost 50 years ago [1]. In humans, pharmacological doses of NA lead to reduction in plasma triglycerides (TG), total cholesterol (TC), low density lipoprotein cholesterol (LDL-C), and an increase in high-density lipoprotein cholesterol (HDL-C). Epidemiological studies have suggested that this improvement in lipid profile can reduce the risk of coronary heart disease [2], through the HDL-C increase, but the recent findings of controlled outcome trials and meta-analyses have not fully supported this hypothesis [3].

Various mechanisms have been reported to explain this HDL-C increase with NA in humans, including enhancement of apolipoprotein AI (apoAI) production but with no change in its fractional catabolic rate [4]; reduction of HDL uptake with no change in cholesteryl ester (CE) uptake, measured *in vitro* [5]; and a reduction of plasma cholesteryl ester transfer protein (CETP) activity, which allows the transfer of TG and CE between HDL and lower density lipoproteins [6,7]. *In vitro* studies have also shown that NA stimulates other pathways involved in HDL metabolism, such as the expression of ATP binding cassette A1 (ABCA1) [8] and peroxisome proliferator-activated receptor (PPAR) γ [9,10], but has no effect on HDL binding, CE selective uptake, or the expression of scavenger receptor class B type 1 (SR-BI) in CHO cells [11]. The ability of NA treatment to increase HDL in humans has not been replicated in animal models. NA treatment affected HDL concentration in transgenic mice expressing human CETP, but not in wild type animals naturally with no CETP activity [7], underlining the key role of this transfer protein.

ApoAI and CE labeling can be used to study the HDL-dependent component of reverse cholesterol transport (RCT). Labeling was first performed with radioactive compounds [12,13], followed by endogenous labeling with stable isotopes [14]. The latter approach is safe and enables the direct assessment of cholesterol esterification rate by lecithin cholesterol acyltransferase (LCAT). This *in vivo* method can be used to study cholesterol flux and to understand the role of CETP in the NA effect. It can be applied in dogs known to have no CETP activity [15], in which RCT is related only to a specific HDL-dependent pathway (11). Moreover, among species used to analyze cholesterol metabolism, dogs exhibit more selective uptake in total HDL-CE turnover [14] than to rats [12,13], mice [16], and humans [17]. Thus, a dog model appears to be a relevant for the examination of HDL metabolism and, notably, *in vivo* modulation of selective CE uptake. Given their size, dogs are well adapted for longitudinal metabolic studies and multiple blood collections. Finally, obese and insulin-resistant dogs exhibit a profile of dyslipidemia (higher TG and lower HDL-C plasma concentrations) [18] observed in patients with metabolic syndrome, known to be partially corrected by NA treatment [19].

The aim of this study was to examine the effects of NA treatment on HDL turnover in obese insulin-resistant dogs. Dual stable isotope infusion was used to assess HDL kinetics through endogenous labeling of cholesterol and apoAI moieties and also to measure cholesterol removal by HDL endocytosis or selective uptake. To assess the effect of NA treatment on ability of serum to promote the cell cholesterol removal, we have also measured the *in vitro* cell cholesterol efflux.

## Materials and Methods

### Animals and treatment

Beagle dogs born in the Reproduction Unit of Oniris (National College of Veterinary Medicine, Food Science and Engineering, Nantes, France) and housed according to the regulations for animal welfare of the French Ministry of Agriculture and Fisheries were enrolled in this study. Only dogs with hematocrit between 37% and 55% and leucocytes between 6.10^9^/L and 17. 10^9^/L, good appetite, no medication use, normal stool, and normal body temperature (38.5–39.5°C) were studied. Experimental protocols adhered to European Union guidelines and were approved by the Animal Use and Care Advisory Committee of the University of Nantes.

Nine male obese insulin-resistant dogs aged 1.8 ± 0.1 years with a mean body weight (BW) of 16.8 ± 0.7 kg were used. Before treatment initiation, dogs were housed together in a 40-m^2^ room in the Nutrition and Endocrinology Unit of Oniris. Body weight and condition were monitored weekly. Obesity was assessed before treatment initiation by fat mass determination (23.7 ± 2.4% fat mass) using a previously described deuterium dilution method [20].

Dogs were fed once daily with a high-fat (32% protein, 20% fat, 4.3 kcal.g^-1^), dry (extruded) commercial diet (Medium Junior; Royal Canin, Aimargues, France). The animals’food intake was recorded daily. Dogs were fed so they can maintain their obese body weight. The daily food amount needed to reach this objective had been previously determined during the 3 weeks before the start of the study. Water was constantly available. During the treatment period, dogs were housed in single cages in the same room, with continued visual contact with each other to prevent isolation stress.

To study the effect on NA treatment on HDL cholesterol turnover, a longitudinal study was performed in six dogs where each dog is its own control. Thus the same dog was studied for all measurement before to start the NA treatment (baseline or week 0) and after 4 week treatment (Week 4). To exclude a drug-independent time effect, three dogs were studied in parallel as controls for clinical and biochemical measurements, including insulin sensitivity evaluation, gene expression, and cholesterol efflux measurement. These 3 dogs did not receive the NA treatment and did not submitted to the kinetic study.

Thus after different measurements at the baseline, the six dogs were given 375 mg.d^-1^ extended-release niacin (NA) (Niaspan; Abbott Laboratories, Abbott Park, IL, USA) for 1 week (23.2 ± 1.2 mg.kg^-1^.d^-1^) and then 500 mg.d^-1^ for 3 weeks (30.9 ± 1.5 mg.kg^-1^.d^-1^), corresponding to a standard treatment in humans (2 g.d^-1^, i.e., 29 mg.kg^-1^.d^-1^). Thus NA was given orally each day before the meal during four weeks. An independent veterinarian monitored the dogs throughout the study period and observed no side effects.

### Euglycemic-hyperinsulinemic clamp

To estimate the insulin sensitivity a 3-h euglycemic-hyperinsulinemic clamp was conducted in 24-h fasted dogs, as previously described [21] in dogs before (week 0) and after (week 4) treatment and in three control dogs. Briefly, human insulin (Actrapid; NovoNordisk, Bagsvaerd, Denmark) was infused *via* the cephalic vein catheter (4 mU.kg^-1^ for 1 min, then 2 mU.kg^-1^.min^-1^ for the duration of the experiment) to induce hyperinsulinemia. Glucose plasma was clamped at basal concentration by adjustment of the glucose (Glucose 20%; Laboratoire Aguettant, Lyon, France) infusion rate in the cephalic vein. Two mL of blood samples were drawn from the jugular catheter every 5 min from 0 to 60 min and every 10 min from 60 to 180 min. Samples were then placed in ice-cold heparinized tubes, centrifuged at 4°C and 2724 *g* for 10 min, and stored at –80°C for further insulin analysis. Rapid determination of blood glucose during clamp was performed using the glucose oxidase method (Glucotrend Plus; Roche Diagnostics, Mannheim, Germany). Plasma insulin concentrations were measured using a commercial radioimmunoassay kit (RIA Insik-5; Diasorin, Saluggia, Italy). The insulin sensitivity index (I_IS_) was defined as the mean glucose infusion rate (mg/kg/min) divided by the mean plasma insulin concentration (μU/mL) during the last 60 min of the clamp [21]. Upon the initial use of the euglycemic-hyperinsulinemic clamp in dogs in our laboratory, we determined that the internal variation was <15%, in accordance with values reported for non-diabetic subjects [22].

### Plasma lipid and lipoprotein analysis

At baseline of the lipid kinetic study, two mL of jugular vein blood samples were collected from 24-h fasted dogs into EDTA tubes (Venoject, Paris, France) and then immediately centrifuged (4°C, 2724 *g*, 10 min) and stored at –80°C for further analysis. Lipoproteins were separated (18) by fast-protein liquid chromatography (GE Healthcare, Pittsburgh, PA, USA). As reported by Kieft *et al*. [23], the precision of this method was determined, using human plasma, by calculating both intra- and interassay precision which is respectively 1.4% and 2.2%. TG, TC, unesterified cholesterol (UC), and unesterified fatty acids (NEFA) were assessed using enzymatic methods (cholesterol RTU and triglycerides enzymatique TG PAP150, Biomerieux, Marcy-l’Etoile, France; free cholesterol FS, Diasys, holzheim, Germany; NEFA C, WAKO, Oxoid, Dardilly, France). Cholesteryl ester (CE) concentration was calculated as TC concentration—UC concentration.

### *In vivo* HDL CE and apolipoprotein kinetics

Two days after the clamp study, lipid kinetic studies were performed using an 8-h constant infusion of [1,2 ^13^C_2_] acetate and [5,5,5 ^2^H_3_] leucine, aprecursors of CE and apoAI, respectively as we have previously described [13] to study the influence of NA treatment on HDL cholesteryl ester turnover in obese insulin resistant dogs. The kinetic experiments were conducted only in NA-treated dogs, before (week 0) and at the end (week 4) of treatment. The last dose of NA was given the morning of the experiment. To realize this kinetic study, two intravenous catheters were inserted: one (Vasocan Braunüle, 20G, 11/4′; B. Braun, Melsungen, Germany) was placed in the cephalic vein of a forelimb for tracer infusion, and another (20G, 8 cm; Vygon, Ecouen, France) was placed in a jugular vein for blood sample collection. Venous blood samples were withdrawn into EDTA tubes (Venoject) at 0, 0.25, 0.5, 0.75, 1, 2, 3, 4, 6, 8, 8.25, 9, 10, 24, and 26 h. Sodium azide (a bacterial growth inhibitor), Pefabloc (protease inhibitor; Interchim, Montluçon, France) and 5,5-dithio-bis-nitro benzoate, an LCAT inhibitor, were added to plasma samples at a final concentration of 1.5, 0.5 mmol/l and 0.2 mmol/L, respectively. Isotopic enrichment of HDL-apoAI and plasma leucine was examined using gas chromatography—mass spectrometry (GC-MS). Lipoproteins were separated by ultracentrifugation and apoAI was isolated from HDL by sodium dodecyl sulfate polyacrylamide gel electrophoresis. ApoAI bands were dried in vacuum for 1 to 2 h (RC 10–10 Jouan, Saint Herblain, France) and hydrolyzed with 1 ml of 4 N HCl (Sigma, St Quentin Fallavier, France) at 110°C for 24 h. The amino acids were purified by cation-exchange chromatography using a Temex 50W-X8 resin (Biorad:Hercules, Calif., USA) and then esterified and derivatized. Electron-impact GC-MS was performed using a 5891A gas chromatograph connected to a 5971A quadrupole mass spectrometer (Hewlett Packard Co., Palo Alto, CA, USA). The isotopic ratio was determined by selected ion monitoring at 282 and 285 m/z.

Analysis of cholesterol and measurement of ^13^C enrichments in UC and CE with gas chromatography—combustion—isotope ratio mass spectrometry (GC-C-IRMS) were performed as described previously [14]. Briefly, plasma lipid extraction was performed with chloroform—methanol (2:1, v/v) to analyze the enrichment in UC and CE. These two forms of cholesterol were first separated by thin- layer chromatography on silicic acid using microcolumns (Sep Pack cartridges; Waters, Milford, MA, USA) [24]. Cholesteryl ester was then saponified in 1 mL ethanol and 200 μL NaOH 10N at 95°C for 1 h to be hydrolyzed. Samples were then cooled at room temperature, and then unesterified cholesterol was extracted two times with 3 mL hexane. The extracts were pooled, evaporated and unesterified cholesterol samples were derivatized with a mixture of acetic anhydride (500 μl) and pyridine (100 μl) (Aldrich, Saint Quentin, France). The samples were heated to 90°C for 10 min. After cooling to room temperature, the derivatizing reagents were evaporated under nitrogen, and the residue was dissolved in hexane. ^13^C Enrichments were measured using GC-C-IRMS with a Finnigan Mat Delta S isotope ratio mass spectrometer coupled to a HP 5890 series II gas chromatograph (Hewlett-Packard, Palo Alto, CA) with a DB-1 capillary column (30 m; 0.32 mm id; 0.25-μm film thickness; J & W Scientific, Rancho Cordova, CA). Results were expressed as Atom Percent Excess as reported in our previous studies [14,17].

**Fig 1:**
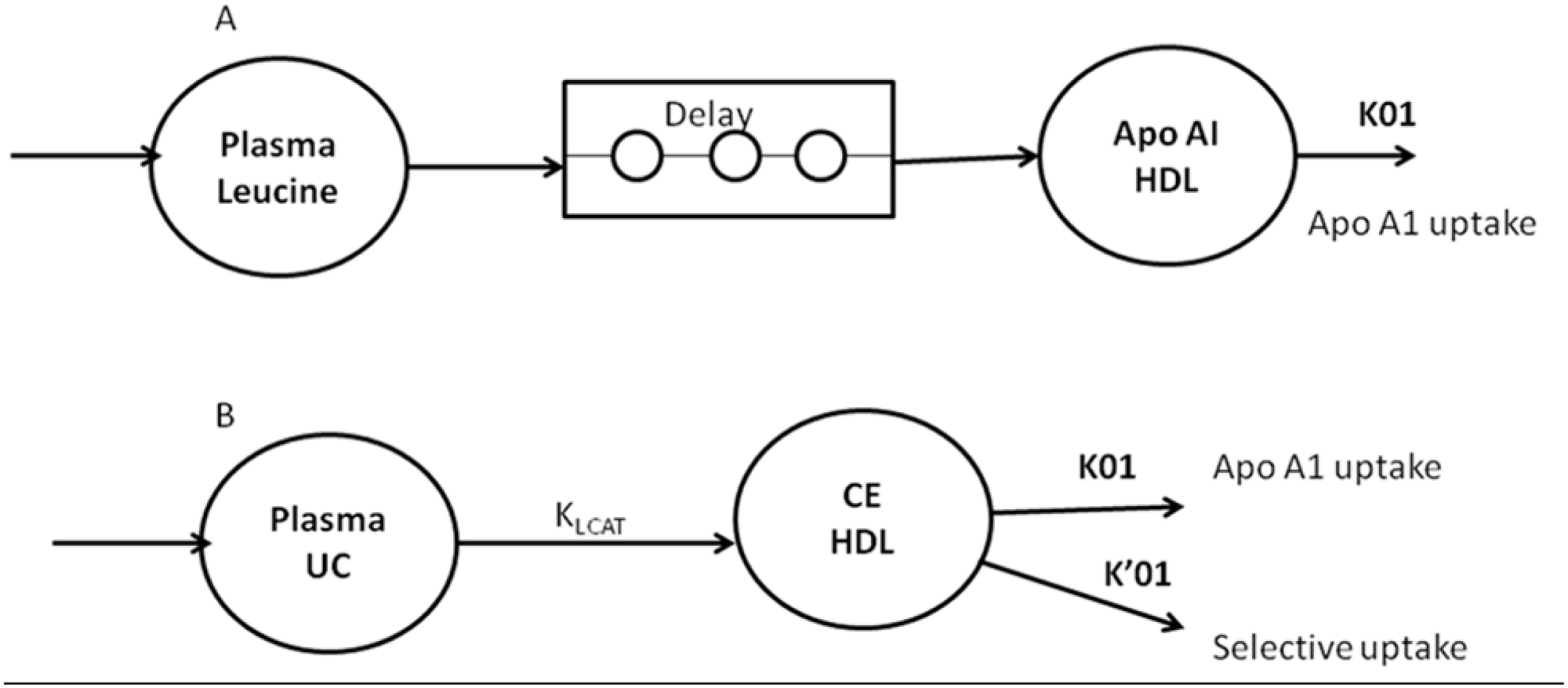
Monocompartmental model used for the modeling of apolipoprotein AI (apoAI) (A) and HDL cholesteryl ester (B) turnover in dogs. UC, unesterified cholesterol; CE, cholesteryl ester; k_01_, apoAI uptake; k_01’_, CE selective uptake; k_LCAT_, cholesterol esterification rate.

Kinetic data analysis was performed with Simulating and Analysis Modeling II software (SAAM Institute, Seattle, WA, USA), as described in humans [17] and dogs [14]. The fate of HDL-CE was considered to be dual: i) simultaneous removal of CE and apoAI through HDL particle uptake and ii) selective CE uptake. To analyze apoAI labeling data, HDL-apoAI was considered in a one-compartment model (Fig 1A). Labeling input was derived after a delay from the plasma-free leucine tracer-to-tracee ratio (as a forcing function). In this model, k01 represented HDL-apoAI endocytosis by tissues, including the liver. To analyze CE labeling data, HDL-CE was also considered in a one-compartment model (Fig 1B). Labeling input was derived from UC enrichment (as a forcing function) through cholesterol esterification (k_LCAT_). In this model, k01 and k’01 represented the two CE outputs, through HDL particle endocytosis by tissues (including the liver) (Fig 1A) and selective hepatic CE uptake, respectively. Parameters were identified simultaneously from HDL-apoAI and CE data. This compartmental analysis provided the fractional catabolic rate (FCR) of apoAI (k01, apoAI catabolism) and selective uptake—mediated CE turnover (k’01, selective uptake). The sum of k01 and k01’ correspond to the total CE FCR. The model also provided the LCAT rate constant (k_LCAT_, in h^-1^), measured as the proportion of plasma UC esterified per hour. All coefficients of variation of calculated model parameters were <5%. The CE or apoAI absolute production rate (APR, in mg.kg^-1^.h^-1^) was defined as the product of the FCR and the pool size of CE or apoAI in HDL. Pools of CE and apoAI in HDL were calculated by multiplying the CE or apoAI concentration by 0.045 (L.kg^-1^), assuming a plasma volume of 4.5% BW [25].

### *In vitro* measurement of cholesterol efflux

To assess the effect of NA treatment on ability of serum to promote the cell cholesterol elimination, we have measured the cholesterol efflux of serum obtained before (week 0) and after NA treatment (week 4) and from controls with a previously described procedure [26,27] using [^3^H] cholesterol—labeled Fu5AH cells. Briefly, Fu5AH cells were cultured in Eagle’s minimum essential medium (EMEM; Sigma, Saint-Quentin Fallavier, France) containing 10% fetal calf serum (Sigma), penicillin, streptomycin, and glutamine. Cells were plated in costar 24-well plates and grown in the appropriate medium at 37°C in a humidified 5% CO_2_ atmosphere. When 80% confluent, cells were incubated at 37°C for 24 h with 1 *μ*Ci.mL^-1^ [1,2-^3^H] cholesterol. To ensure that the label was distributed evenly among cellular pools, the labeling medium was replaced with EMEM containing 0,1% bovine serum albumin (Sigma), and cells were incubated for 18–20 h before cholesterol efflux was measured. The cells were then washed and incubated with the different sera from dogs (control dogs and dogs before (week 0) and after NA treatment (week 4)) prepared in EMEM (5% [vol/vol], total volume was 500 μl), and efflux was performed for 4 h. After the efflux period, media were collected and radioactivity was determined by liquid scintillation counting. The residual radioactivity in the cells was determined after extraction with 500 μl of isopropanol. The percent efflux was calculated by dividing the radioactivity counts in the media by the sum of the radioactivity counts in the media plus the cell fraction. We conducted multiple determinations using a single human serum and found an acceptable intra-assay reproducibility (n = 10, mean CV 8% ranged from 7.25 to 9%), as reported previously [27].

### Hepatic mRNA expression

To measure the expression of key genes (Table 1) involved in HDL cholesterol metabolism, liver biopsies were performed twice under anesthesia following 24-h fasts before (week 0) and at the end of the 4-week treatment (week 4). Biopsy forceps are used to remove small amounts (approximately 100 mg) of liver from the left lobe (lateral and median parts). All biopsies have been taken by the same surgeon. Dogs were anesthetized with a combination of medetomidine (Domito; Pfizer, Paris, France), butorphanol (Dolorex; Intervet, Beaucouzé, France), and ketamine (Imalgène; Merial, Villeurbanne, France), mixed in one syringe and administered intravenously in a single dose. After endotracheal intubation, anesthesia was maintained with 1.5% isoflurane. Dogs were monitored by clinical observation, respiratory rate measurement, temperature measurement, electrocardiography, pulse oximetry, and capnography. No medication was used to wake the dogs.

**Table 1:** Sense/antisens primers used for ABCA1, SR-BI, PPARα, ATP synthase β chain and GAPDH relative quantification. (PPARα: peroxisome proliferator-activated receptor α, ABCA1: ATP binding cassette A1, SR-BI: scavenger receptor class B type I, GAPDH: glyceraldehyde-3-phosphate dehydrogenase).

Gene	Primers sense/antisense
ABCA1	5’-TGGACAGCAGAAGCAATGAC-3’
	5’-TAAGCCGACTTCTGTTGCT-3’
SR-BI	5’-AGGGCAAGTTTGGGCTATTT-3’
	5’-GAATTCCAGCGAGGTCTCAG-3’
PPARα	5’-TTATCACAGACACGCTCTCACC-3’
	5’-GTGGACTCCGTAATGGTAGC-3’
ATP synthase	5’-ACCATTGAAGAAGCTGTGG-3’
	5’- CATGTGGCCTACAGAGCAA-3’
GAPDH	5’-ACAGTCAAGGCTGAGAACGG-3’
	5’-CCACAACATACTCAGCACCAGC-3’

Tissue was cleaned in saline. TRIzol reagent (Invitrogen, Carlsbad, CA, USA) was added and the tissue was frozen immediately in liquid nitrogen. RNA was extracted from frozen tissue using TRIzol reagent according to the manufacturer’s instructions. Total RNA concentration was quantified by spectrophotometric absorbance at 260 nm. The 260-to-280-nm absorption ratios of all samples were between 1.8 and 2.0.

Total RNA (1 μg) was reverse transcribed in a 20 μL reaction volume using random primers (Pharmacia, Saclay, Orsay Cedex, France) and superscript II reverse transcriptase (Life Technologies, Cergy Pontoise, France) according to the manufacturer’s instructions. Real-time polymerase chain reaction (PCR) analysis was performed using a 7000 Sequence Detection System with SYBR green PCR Master Mix (Eurogentec, Angers, France). Sense/antisense primer sequences (Genosys, Pampisford, UK) for ABCA1, SR-BI, PPARα, ATP synthase β chain, and glyceraldehyde-3-phosphate dehydrogenase (GAPDH) were designed using the web-based Primer 3 program (http://frodo.wi.mit.edu/primer3/) and are listed in Table 1. Relative quantitative expression levels were normalized using GAPDH as a housekeeping gene and were calculated using the 2^-ΔΔt^ method [28]. The level of gene expression before NA treatment was arbitrarily set to 100%. The coefficient of variation of gene expression measurement in our study was less than 6% as reported previously [29].

### Statistical analysis

Data are expressed as medians with interval ranges. The statistical analysis of kinetic and gene expression results was performed to compare obese dogs at baseline (week 0) and after 4 weeks of treatment (week 4) using the nonparametric Wilcoxon matched-pairs signed rank test with StatView 5.0 software (SAS Institute, Cary, NC, USA), due to limited group size, which prevented us from ensuring the normal distribution of the data. *P* values < 0.05 were considered to be significant.

To compare NA effects between groups, repeated-measures analysis of variance in plasma lipid and sensitivity index (I_IS_) parameters was performed using a linear mixed-effects model (S-Plus software, version 6.2; TIBCO Software Inc, Somerville, MA, USA). *P* values < 0.05 were considered to be significant.

## Results

### Body weight and insulin sensitivity

Compared to baseline (week 0) NA treatment during four weeks did not affect the dogs body weight (Table 2). As treatment with NA was reported to affect insulin sensitivity [30–32], we measured the index of insulin sensitivity (I_IS_) at baseline (week 0) and after four weeks of treatment (week 4) by using euglycemic hyperinsulinemic clamp. Extended-release niacin (NA) did not change significantly the I_IS_ between week 0 and week 4.

**Table 2:** Body weight (BW), insulin sensitivity index (I_IS_), fasting plasma triglycerides (TG), total cholesterol (TC), unesterified cholesterol (UC), cholesteryl ester (CE), unesterified fatty acid (NEFA), VLDL-triglycerides (VLDL-TG), LDL-cholesterol (LDL-C), HDL-cholesterol (HDL-C), HDL-phospholipids (HDL-PL) in control dogs (n = 3) and in NA-treated dogs (n = 6) before (week 0) and at the end of the treatment (week 4). Values are presented as median [minimum-maximum]. NS, non significant.

	Control dogs		NA treated dogs		*p* (NA effect)
	Week 0	Week 4	Week 0	Week 4	
**BW (kg)**	15.2 [12.7–18.3]	14.7 [12.6–17.8]	16.2 [15.2–20.5]	16.5 [14.7–19.15]	NS
**IIS**	0.14 [0.09–0.20]	0.10 [0.05–0.13]	0.12 [0.06–0.17]	0.06 [0.04–0.10]	NS
**TG (mmol.L-1)**	0.97 [0.78–0.87]	0.89 [0.72–1.01]	0.94 [0.71–1.26]	0.59 [0.46–0.94]	< 0.05
**TC (mmol.L-1)**	4.98 [4.92–5.51]	4.76 [3.97–4.91]	4.78 [4.04–5.07]	3.44 [2.84–3.87]	< 0.05
**UC (mmol.L-1)**	0.84 [0.95–0.77]	0.86 [1.01–0.74]	0.83 [0.77–0.88]	0.83 [0.73–0.93]	NS
**CE (mmol.L-1)**	4.02 [3.93–4.19]	3.87 [3.61–4.05]	3.88 [3.23–4.20]	2.55 [2.07–3.02]	< 0.05
**NEFA (mmol.L-1)**	0.77 [0.74–1.10]	0.87 [0.81–1.00]	0.93 [0.61–1.13]	1.22 [0.75–1.23]	NS
**VLDL-TG (mmol.L-1)**	0.03 [0.02–0.14]	013 [0.02–014]	0.15 [0.10–0.25]	0.10 [0.03–0.14]	< 0.05
**LDL-C (mmol.L-1)**	0.34 [0.23–0.36]	0.29 [0.17–0.39]	0.38 [0.29–0.64]	0.19 [0.16–0.39]	< 0.05
**HDL-C (mmol.L-1)**	4.30 [4.55–0.36]	4.14 [3.73–4.17]	4.08 [3.51–4.42]	2.92 [2.45–3.36]	< 0.05
**HDL-PL (mmol.L-1)**	1.24 [1.18–2.00]	1.92 [1.00–2.12]	1.33 [0.90–1.54]	1.41 [1.34–1.87]	NS

To assess a drug-independent time effect on these parameters three control dogs were studied and Body weight and I_IS_ remained constant during study.

### Plasma and lipoproteins lipids

The effect of NA in the dogs before treatment (week 0) and after treatment (week 4) on plasma lipids is shown in Table 2. NA treatment reduced plasma triglycerides (TG) (-32%, p<0.05) and plasma total cholesterol (TC) or cholesteryl ester (CE) to the same magnitude (-27%; both *p* < 0.05) whereas unesterified cholesterol (UC) was unchanged. No difference was observed in plasma non esterified fatty acids (NEFA) concentrations with NA treatment.

Lipoproteins were separated using fast protein liquid chromatography and data are shown in Table 2. VLDL TG were reduced (-44%, p<0.05) by NA treatment. Plasma LDL-C and HDL-C were also reduced (-43% and -27%, respectively; both *p* < 0.05) after treatment, whereas HDL phospholipids were not changed.

All these parameters remained stable during study in the three control dogs.

### ApoAI and cholesterol kinetics studies

[1,2 ^13^C_2_] acetate and [5,5,5 ^2^H_3_] leucine during 8h in dogs before NA treatment (week 0) and four weeks (week 4) after NA treatment to label the HDL apoAI and cholesterol respectively to assess the effect of this drug on HDL CE turnover.

The concentrations of cholesterol and apoAI were measured before and 3 sampling times during isotope infusion. Because no significant variations were observed between measurements, all dogs were considered in metabolic steady state throughout the stud. Kinetic parameters data are presented in Table 3. All kinetic parameters coefficients of variation are less than 5% (data not shown). NA treatment reduced the HDL-ApoAI concentration (13%, *p* < 0.05), in agreement with an increase in fractional catabolic rate (FCR = k01) of ApoAI (210%, *p* < 0.05) that was not compensated by an increase in absolute production rate APR of ApoAI (170%, *p* < 0.05). The in vivo esterification rate assessed by K_LCAT_ measurement and the CE APR were increased after NA treatment (week 4) compared to basal state (week 0) (both ~40%, *p* < 0.05), whereas the HDL-CE concentration was reduced (32%, *p* < 0.05) due to a higher total HDL-CE FCR (= k01+k01’) (107%, *p* < 0.05). This increased catabolism was explained by greater selective uptake (108%, *p* < 0.05) and a higher apoAI FCR.

**Table 3:** Kinetic parameters of HDL apolipoprotein AI and HDL-cholesteryl ester before (week 0) and after 4 weeks of NA treatment (week 4) identified using models of Fig 1. FCR, fractional catabolic rate; APR, absolute production rate; HDL-UC, HDL unesterified cholesterol concentration; HDL-CE, HDL cholesteryl ester concentration; K_LCAT_, cholesterol esterification rate by LCAT. Data are expressed as median [minimum-maximum], n = 6.

	Week 0	Week 4	*p*
**HDL-ApoAI (g.L** ^**-1**^ **)**	0.93 [0.91–1.03]	0.80 [0.73–1.03]	< 0.05
**APR ApoAI (mg.kg** ^**-1**^ **.h** ^**-1**^ **)**	0.267 [0.134–0.298]	0.368 [0.240–0.710]	< 0.05
**FCR ApoAI (k**_**01**_ **, h** ^**-1**^ **)**	0.0065 [0.0032–0.0071]	0.0103 [0.0062–0.0200]	< 0.05
**HDL-CE (g/L** ^**-1**^ **)**	1.34 [1.11–1.45]	0.88 [0.71–1.04]	< 0.05
**APR HDL-CE (mg.kg** ^**-1**^ **.h** ^**-1**^ **)**	1.51 [1.11–2.95]	2.09 [1.91–3.56]	< 0.05
**FCR HDL-CE (h** ^**-1**^ **)**	0.025 [0.021–0.059]	0.055 [0.41–0.111]	< 0.05
**K**_**LCAT**_ **(h** ^**-1**^ **)**	0.101 [0.079–0.209]	0.154 [0.126–0.266]	< 0.05
**Selective uptake (k’**_**01**_ **, h** ^**-1**^ **)**	0.020 [0.014–0.052]	0.045 [0.029–0.092]	< 0.05

### *In vitro* cholesterol efflux and hepatic gene expression

To assess the effect of NA treatment on ability of serum to promote the cell cholesterol removal, we have measured the cholesterol efflux of serum obtained before (week 0) and after NA treatment (week 4) and from controls. Sera from NA-treated dogs (week 4) induced significant increase of [^3^H]-cholesterol efflux (16%; from 37.3% [32.8%-45.3%] to 43.4% [37.5%-50.5%]; *p* < 0.05) compared to basal state (week 0). Cholesterol efflux did not change in the three control dogs between week 0 and week 4 (36,1 [30.3–47.0] *vs*. 39.0 [29.8–54,9].

**Fig 2:**
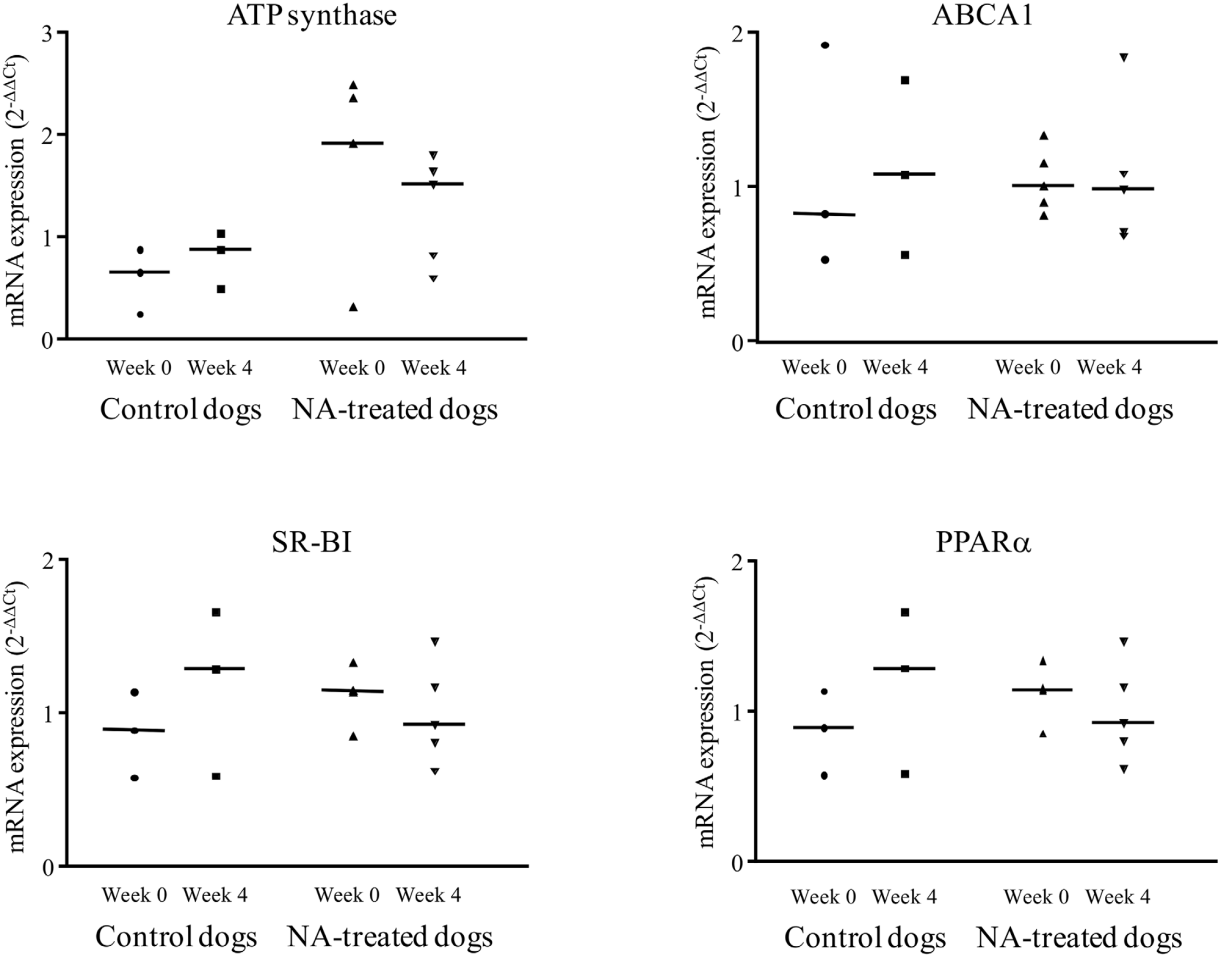
Relative expression of PPARα, SR-BI, ABCA1 and ATP synthase mRNA in liver of NA treated dogs, before (week 0) and after the end of the 4 weeks treatment (week 4). (PPARα: peroxisome proliferator-activated receptor α, ABCA1: ATP binding cassette A1, SR-BI: scavenger receptor class B type I), n = 6.

Compared with basal values obtained at week 0, the hepatic mRNA expression of PPARα, ABCA1, ATP synthase β chain, and SR-BI were unchanged after four weeks of NA treatment (Fig 2). No change in mRNA expression was observed in the control group.

## Discussion

Insulin resistance is associated with so-called “atherogenic dyslipidemia,” and extended-release niacin (NA) can correct these disturbances. The current study analyzed the HDL cholesterol turnover effect of NA using dual stable-isotope endogenous labeling of HDL-C and apoAI and modeling analysis. NA treatment had no effect on the I_IS_, which was measured in the present study because NA has been reported to increase the risk of diabetes onset [3,30–33]. In our study, we found that NA treatment improved reverse cholesterol transport (RCT) by increasing cholesterol esterification, HDL cholesterol disappearance through CETP independent pathways (i.e both endocytosis and selective uptake) and cell cholesterol efflux.

Endogenous labeling achieved rapid equilibration between lipoprotein fractions for unesterified cholesterol (UC) enrichment in this study (data not shown), as reported previously in dogs [14] and humans [17]. This observation demonstrates the rapidity of UC exchanges between lipoproteins and provides an argument for the use of plasma UC as a precursor pool.

In this study we showed that NA treatment significantly enhanced esterification activity (k_LCAT_) and cholesteryl ester (CE) appearance in HDL. The cholesterol esterification in HDL by LCAT lead to cell cholesterol efflux which is the first critical step of the RCT pathway and the mechanism by which macrophages in the vessel wall secrete cholesterol outside cell. The increase of cholesterol esterification we measured in this study suggests a greater effectiveness of peripheral cell cholesterol efflux [34] and then an antiatherogenic role [35]. This *in vivo* result is in accordance with our observation of the greater efficiency of serum from NA-treated dogs in promoting cholesterol efflux in Fu5AH cells. This cell line was selected because it has shown direct binding of HDL [36] with reproducible cholesterol efflux measurement [26,27]. Previously reported data from hypercholesterolemic rabbit adipocytes treated with NA indicate increased cholesterol efflux, related to stimulated ABCA1 pathway [10]. In the present study we measured an unchanged ABCA1gene expression with NA treatment. This result conflicts with data from other studies [8,37,38], probably related to the use of different target tissues.

The significant reduction in HDL-cholesteryl ester (HDL-CE) achieved by NA treatment is related to a higher fractional catabolic rate (FCR), linked to enhancement of apoAI-dependent and selective CE uptake which is attributed to SR-BI receptor. We measured an unchanged hepatic mRNA SR-BI expression by NA treatment in agreement with previous findings [11]. These conflicting results (higher HDL-CE catabolism but unchanged SR-BI expression) suggest that NA affects other pathways involved in CE turnover and selective uptake, such as transintestinal cholesterol excretion [39]. Further studies are needed to assess the effect of NA treatment on this pathway. We also measured an unchanged hepatic expression of ATP synthase β chain (P2Y13), another reported HDL receptor [40].

Kinetic studies examining the effects of NA on HDL in humans are scarce, and no study has examined cholesterol turnover. One study involving two healthy subjects found lower HDL apoAI catabolism [41], whereas another study involving five healthy subjects showed no change [42]. More recently, a higher apoAI-APR with no change in catabolic rate was reported in five patients with combined hyperlipidemia [4]. The reduction in HDL-C and apoAI related to NA in dogs is the main difference from NA’s effects in humans, although TG reduction is similar in both species. In mice, another CETP-devoid species, NA treatment causes no change in HDL-C, despite plasma TG reduction [7]. These studies in two animal species with no CETP activity demonstrated that NA’s benefits with regard to dyslipidemia and, in turn, the cardiovascular system were not dependent on changes in HDL-C, as reported in a recent meta-analysis [3].

We [43] and Rader DJ et al, [44] have shown that the HDL-apoAI FCR is correlated directly with plasma TG through CETP activity. To date, most studies have shown that CETP plays a key role in the effect of NA [45], leading to increased HDL-C and apoAI in humans. Given this important role [17,46], the preponderance of CETP-mediated HDL-C elevation may have hidden potentially greater HDL-CE turnover in humans. Our study showed that NA also accelerates HDL turnover in dogs, revealing the role of other mechanisms. Compared with mice, dogs presented active RCT *via* high LCAT activity followed by efficient selective HDL-CE uptake independent of apoAI catabolism [14], and the HDL turnover enhancement we have described may have been easier to detect in this species. Thus, we can speculate that NA acts through two pathways in species with CETP: i) by elevating HDL-C as a result of a lowered plasma TG concentration through the reduction of CE transport toward diminished TG-rich lipoproteins and ii) by lowering HDL as a result of a higher HDL FCR, which would reflect more active RCT. A study examining these pathways in humans is warranted to confirm this hypothesis.

In conclusion, this study showed that obese insulin-resistant dogs treated with NA reduced HDL-C by raising the catabolic rate secondary to higher HDL endocytosis and selective HDL-CE uptake. NA treatment increased esterification rate and cholesterol efflux, key processes in RCT. These results suggest that NA treatment could improve RCT in humans and demonstrate the relevance of further kinetic studies using endogenous cholesterol labeling in humans.

## Abbreviations

ABCA1: ATP binding cassette A1
apoAI: apolipoprotein A1
APR: absolute production rate
BW: body weight
CE: cholesteryl ester
CETP: cholesteryl ester transfer protein
EMEM: Eagle’s minimum essential medium
FCR: fractional catabolic rate
GAPDH: glyceraldehyde-3-phosphate dehydrogenase
GC-MS: gas chromatography—mass spectrometry
GC-C-IRMS: gas chromatography—combustion—isotope ratio mass spectrometry
HDL-C: high-density lipoprotein cholesterol
I_IS_: insulin sensitivity index
LCAT: lecithin cholesterol acyltransferase
LDL-C: low-density lipoprotein cholesterol
NA: extended-release niacin
NEFA: unesterified fatty acids
PCR: polymerase chain reaction
PPAR: peroxisome proliferator-activated receptor
RCT: reverse cholesterol transport
SR-BI: scavenger receptor class B type 1
TC: total cholesterol
TG: triglycerides
UC: unesterified cholesterol
